# Spatial–temporal trends of COVID-19 infection and mortality in Sudan

**DOI:** 10.1038/s41598-022-21137-z

**Published:** 2022-10-07

**Authors:** Ghada Omer Hamad Abd El-Raheem, Hind Eltayeb Salih Elamin, Zuhal Mohammednour Omer Ahmad, Mounkaila Noma

**Affiliations:** 1 Clinical Pharmacy, High Diploma in Research Methodology and Biostatistics, American Board Certified Critical Care Clinical Pharmacist, Soba University Hospital, National University, Khartoum, Khartoum 00000 Sudan; 2 Royal College of Physicians in London, Research Methodology, Harvard University, Abu Dhabi Telemedicine Centre, P.O. Box 147722, Abu Dhabi, United Arab Emirates; 3 Emergency Department, Imperial Hospital, Alsharif Alhindi Street, Khartoum, Khartoum 00000 Sudan; 4 University of Medical Sciences and Technology, Mecca Street, P.O. Box 12810, Khartoum, Sudan

**Keywords:** Health care, Health occupations, Medical research

## Abstract

Since its emergence, the coronavirus disease 2019 (COVID-19), is constantly affecting many parts of the globe and threatening millions of lives worldwide. Charting and aligning disease incidence to identify spatial clustering and patterns continue to be a substantial pathway to understanding disease epidemiology and is essential for implementing effective planning and prevention strategies. A national descriptive study was implemented to present the infection and mortality rates of the COVID-19 pandemic in all states of Sudan. Data were collected and summarized in monthly statistical reports of COVID-19 infection and mortality rates. The reports used were from May 2020 to March 2021. The highest COVID-19 incidence rate occurred in December 2020 with a total incidence of 4863 cases ranging from 0 cases in some of the states to 4164 cases in other states (mean = 270 ± 946, median = 21 cases). Followed by the incidence in May 2020 with a total of 4524 cases ranging from 4 to 3509 cases (mean = 251 ± 794, median = 31 cases). The western and southern states of the country had the lowest mortality rates. While, the middle states (Khartoum and El Gezira) had the highest mortalities. Northern and eastern states had lower mortalities than the middle states, yet, higher than the western states. A strong positive correlation between infection and mortality was found.

## Introduction

The coronavirus disease 2019 (COVID-19) had been announced as a pandemic by the World Health Organization (WHO) on 11 March 2020^1^. Since its emergence, COVID-19 had affected many parts of the globe and threatened millions of lives worldwide and became a major threat to public health. COVID-19 had massively impacted economies, the environment, and the everyday lives of people around the world^2^. Analysis of the global effect of COVID-19 have substantially portrayed how this disease managed to impact nearly 150 countries/territories/areas over just two months^3^. Since the first cases of COVID-19 were documented in Wuhan, China, countries, and health authorities have responded extensively but differently to the pandemic^4–6^. Unfortunately, each country in the world was combating and dealing with the crisis based on its resources, possibilities and expertise due to the lack of a unified global standard response to the pandemic^7^. Several factors were related to the distribution of COVID-19. Among these factors, the sociodemographic factors; such as, old age, illiteracy, population density, and urbanization rate. Moreover, climate-related factors such as humidity, and solar radiation had an inverse correlation to the infection rate. On the other hand, population density and movement had a direct correlation to the infection rate of COVID-19^8^. In Africa, the inadequacy of healthcare systems and lack of funding were considered as major causes of concern for global experts regarding the spread, and control of COVID-19. Early detection, prevention, and control of the outbreak was defective and inadequate in Africa for reasons related to poor disease surveillance, insufficient data transmission, and unsatisfactory healthcare workers’ training^9–11^. According to the WHO, over 278 million confirmed cases and just under 5.4 million deaths have been reported worldwide by 26 December 2021. The awareness of the epidemiological and geographical characteristics of COVID-19 had become particularly crucial in controlling the spread of the pandemic. The selection of proper measures for understanding and explaining the spread of this disease in order to model the spread of COVID-19 are yet to be identified^12^. A significant number of statistical models were applied for the prediction of the infection rate and the spread of cases during the pandemic^13^. However, charting and aligning of disease incidence to identify spatial clustering and patterns continued to be a substantial pathway for understanding the disease epidemiology, and implementing effective prevention strategies^14^. Till the 31st of December 2020, African countries had cumulative COVID-19 cases of 2,763,421 which represented (3.4%) of the total cases (82,312,150). While, the cumulative mortality was 65,602 deaths, representing 3.6% of the total 1,798,994 deaths worldwide^15^. The challenges for the African countries in order to respond to the COVID-19 pandemic were detailed by Margolin et al.^16^. The limited testing capacity, the relatively poor healthcare systems, the lack in the pharmaceutical industry and manufacturing, as well as the underdeveloped infrastructure in African countries were factors needed to be addressed in order to optimize the response of Africa to the COVID-19 pandemic^16^. The spatial–temporal presentation of the pandemic in Africa was presented at country level^17^. In sub-Saharan Africa, the countries with high infection rate of COVID-19 were Kenya, Ghana, Nigeria, Ethiopia, and South Africa. While, the Seychelles, Eritrea, Mauritius, Comoros, and Burundi had the lowest infection rate^18^. Sudan has the third largest area of any country in Africa and it is considered exposed to the dissemination of infectious diseases, including COVID-19. In Sudan, the first reported COVID-19 case was in March 2020 based on the reports of the "Federal Ministry of Health". The experience of Sudan with COVID-19 was similar to other sub-Saharan countries, the infection rate and the mortality rate have had a peaky nature. The reported cases of COVID-19 in Sudan since the beginning of the pandemic were mainly reported through the Federal Ministry of Health which might reflect an underestimated situation. Sudanese population had limited isolation centers; only Khartoum state had a relatively better healthcare system. Most of the Sudanese population had to travel to Khartoum State to get proper care. This had affected the capacity of healthcare services in Khartoum State and many people treated their patients at home by providing oxygen at home and getting home nursing. In Sudan, from the 3rd of January 2020 until the 15th of September 2022, there have been 63,275 confirmed COVID-19 cases and 4,961 COVID-19 deaths as reported by the World Health Organization^19^. However, a detailed study about the infection and mortality status in Sudan through time; especially at states level was not discussed. This study aimed to assess the epidemiological and spatiotemporal patterns of COVID-19 in Sudan and to draw attentions to strategies for appropriate distribution of the healthcare resources to impede the progress of the pandemic. The rational of this study was to detail the rates of infection and mortality based on the space and time in order to give a clear picture about the places (states) of increased infection, as well as, the seasons (months). This study might be a good reference to the concerned facilities to enlighten them about the areas with high needs, for considering vaccination distribution and healthcare provision. The presented results can help interpreting, and studying the common spatial–temporal trends and patterns of COVID-19 in order to provide a rapid source of information to the concerned facilities.

## Materials and methods

A national descriptive study was implemented to present the infection and mortality rates of COVID-19 pandemic in all states of Sudan. Based on the reports of the Federal Ministry of Health in Sudan (http://sho.gov.sd/corona/), data were collected and summarized in a monthly statistical reports of COVID-19 infection and mortality rates. The reports used were from May 2020 till March 2021. Geographic information system (ArcGIS); ArcGIS version 10.8 was used to develop the maps. The .shp file of the Sudan base map was obtained from the open access organization DIVA-GIS (https://www.diva-gis.org/gdata). The statistical package for social sciences (SPSS version 23) was used to assess correlation between infection rate and mortality rate. It was considered statistically significant when p ≤ 0.05. Ethical approval from the office of the Minister of the Federal Ministry of health was obtained firstly, then the approval from Directorate General of Planning and Health Development was granted on October 14th, 2021. Our study involved collecting the reports that were already decimated by the federal ministry of health. The researchers had no clinical contact to any cases, and only the statistical reports of the ministry of health were used. The collected data were used strictly for the purpose of this study objectives. The reports were publicly published in the Sudan Federal Ministry of Health website (http://sho.gov.sd/corona/). Reports were only statistical with no personal data. The directorate general of emergency and epidemic control at the federal ministry of health had announced public health emergency on March 2020 and asked all Sudanese citizens to immediately report suspected cases through the hotlines 221, 9090. All cases were reported based on their willingness and no identifiers were used during the collection of the data, data were collected as statistical reports only. Signed informed consents were obtained from each case prior to collecting the samples for COVID-19 testing as per the national ethical guidelines. All methods were performed in accordance with the relevant guidelines and regulations of the Declarations of Helsinki. Furthermore, signed informed consents were obtained from the surrogate decision makers regarding the mortality reports of the cases. The research office of the federal ministry of health (research.dep.fmoh@gmail.com) is the official department concerned with the ethical clearances. Reports of COVID-19 cases and mortality were issued by the directorate general of emergency and epidemic control after being signed and stamped by the general director office.

## Results

### Description of COVID-19 infection rate in Sudan

COVID-19 infection in Sudan had wide variation during the study period across the different states of Sudan. The highest incidence rate had occurred in December-2020 with a total incidence of 4863 cases ranging from 0 cases in some of the states to 4164 cases in other states (mean = 270 ± 946, median = 21 cases). Followed by the incidence in May, 2020 with a total of 4524 cases ranging from 4 to 3509 cases (mean = 251 ± 794, median = 31 cases). Table 1 below illustrates the incidence rate of COVID-19 in Sudan.

**Table 1:** COVID-19 infection rate in Sudan.

Infection/months	Min.–max	Mean ± SD	Median	Total
May-20	4–3509	251 ± 794	31	4524
Jun-20	1–3001	249 ± 679	74	4486
Jul-20	0–1287	116 ± 291	14	2093
Aug-20	0–1099	74 ± 250	1	1340
Sep-20	0–360	26 ± 82	2	462
Oct-20	0–125	9 ± 28	1	165
Nov-20	0–3663	235 ± 834	14	4238
Dec-20	0–4164	270 ± 946	21	4863
Jan-21	1–2233	201 ± 503	53	3609
Feb-21	0–669	53 ± 151	10	955
Mar-21	0–1145	94 ± 261	13	1683
Cumulative	10–21,255	1579 ± 4801	344	28,418

### Spatial–temporal COVID-19 infection across the states of Sudan

**Figure 1:**
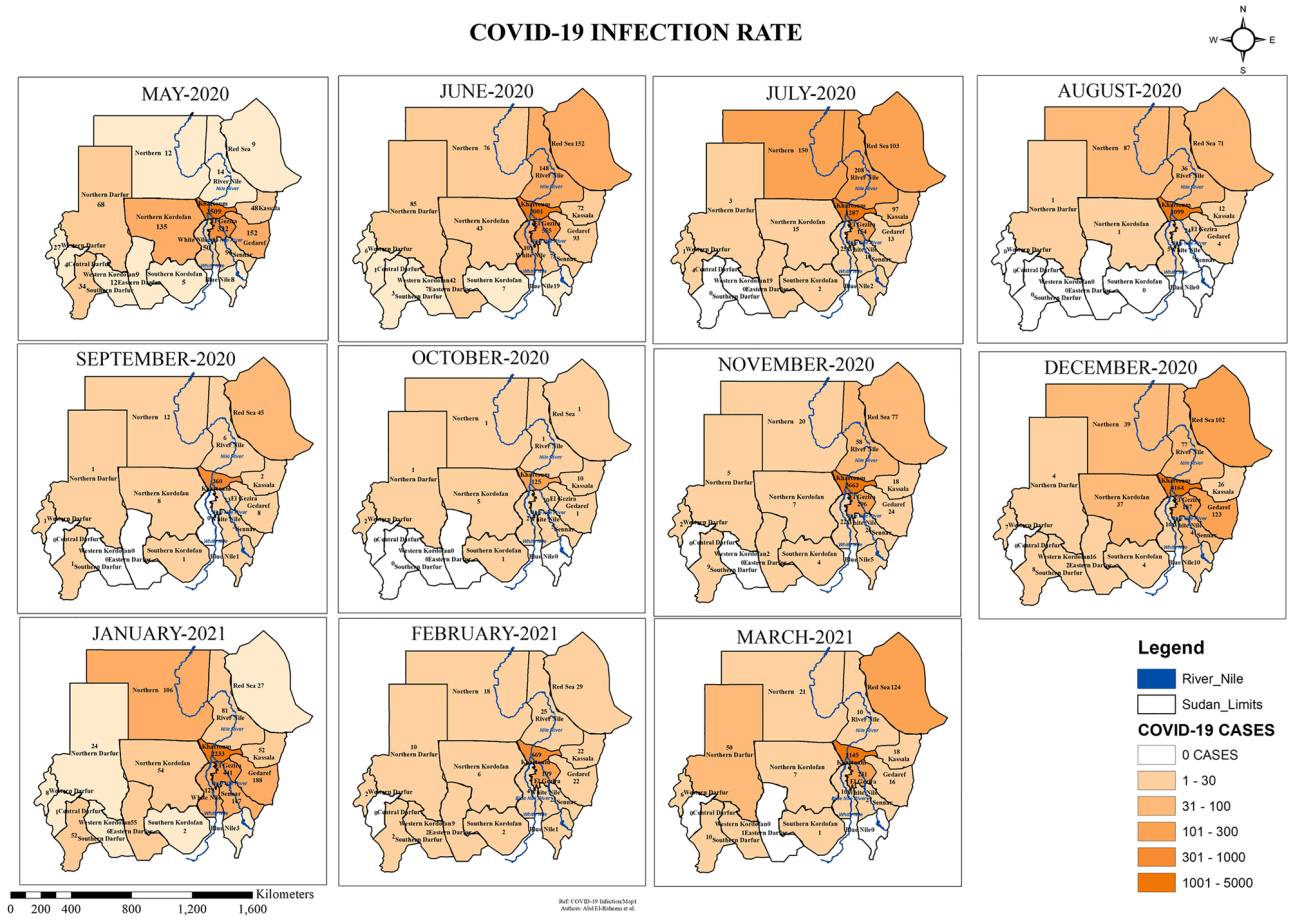
COVID-19 Infection rate across the states of Sudan May 2020–March 2021 developed through ArcGIS 10.8 by Abd El-Raheem et al. (Shp. files of the base maps https://www.diva-gis.org/gdata).

The incidence rate of COVID-19 across the different states of Sudan was studied (Fig. 1). The spatial–temporal trend of this pandemic varied across different states. Khartoum State was the most affected state during all the months, followed by El Gezira State. Both of these states lay in the middle of the country. The western states of the country had lower incidence rate of COVID-19 infection. October, 2020 had the lowest incidence rate in all states. Furthermore, five states had zero incidence of COVID-19 infection in October, 2020. The wavy nature was apparent in the different states across the months. Figure 1 below shows the spatial–temporal presentation of COVID-19 infection.

### Correlation between cumulative COVID-19 infection and population density across the States of Sudan

**Figure 2:**
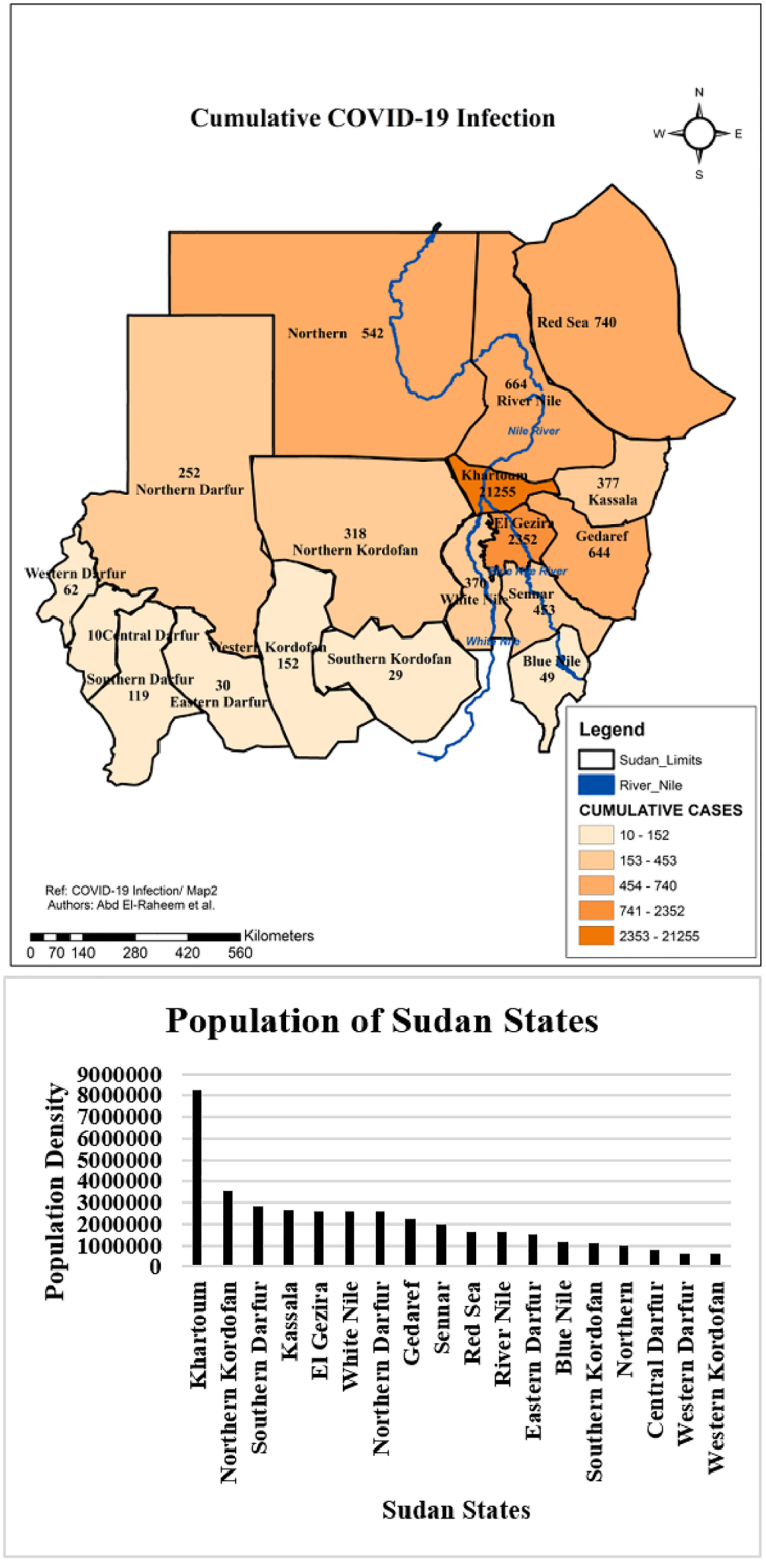
Cumulative COVID-19 Infection across the Sudan States and population of each State developed through ArcGIS 10.8 by Abd El-Raheem et al. (Shp. files of the base maps https://www.diva-gis.org/gdata).

On assessing the correlation between COVID-19 infection and the population density in each state, a strong positive correlation (Pearson’s correlation = 0.885, p-value = 0.000) was found between cumulative infection and population density across Sudan States. The middle states (Khartoum and El Gezira) had the highest infection rate and the highest population density (Fig. 2). While, the western states (Western Darfur, Central Darfur and Eastern Darfur) had the lowest rate of COVID-19 infection. These western states had low population density (Fig. 2).

### Description of COVID-19 mortality rate in Sudan

Mortality rate among COVID-19 cases had wide variations, yet it had less variability than the infection rate. During all of the months, some of the states had zero mortalities. Nevertheless, the rate of mortality showed similar pattern of the wavy nature. These surges of COVID-19 were apparent, the peaks of mortality rate were during May and June -2020, with total mortalities of 295 (mean = 16 ± 39) and 301 (mean = 17 ± 30) cases respectively. Then another surge was apparent in December-2020 and January-2021, with mortalities of 192 (mean = 11 ± 37) and 191 (mean = 11 ± 29) cases respectively. As well as, another surge was starting in March-2021, which showed mortality rate of 181 cases (mean = 10 ± 24 cases). The cumulative mortality of COVID-19 during the 11 month of the study period was 1565 cases with a mean of 87 ± 188 cases (median = 29 cases). Table 2 below illustrated the statistics of mortality for each month.

**Table 2:** COVID-19 mortality rate in Sudan.

Mortality/months	Min.–max	Mean ± SD	Median	Total
May-20	0–167	16 ± 39	2	295
Jun-20	0–115	17 ± 30	6	301
Jul-20	0–33	8 ± 11	2	146
Aug-20	0–15	4 ± 5	0	69
Sep-20	0–5	1 ± 1	0	15
Oct-20	0–1	0 ± 0	0	1
Nov-20	0–84	5 ± 19	0	94
Dec-20	0–164	11 ± 37	1	192
Jan-21	0–110	11 ± 29	0	191
Feb-21	0–22	4 ± 6	2	80
Mar-21	0–100	10 ± 24	2	181
Cumulative	1–801	87 ± 188	29	1565

### Spatial–temporal COVID-19 mortality rate across the States of Sudan

**Figure 3:**
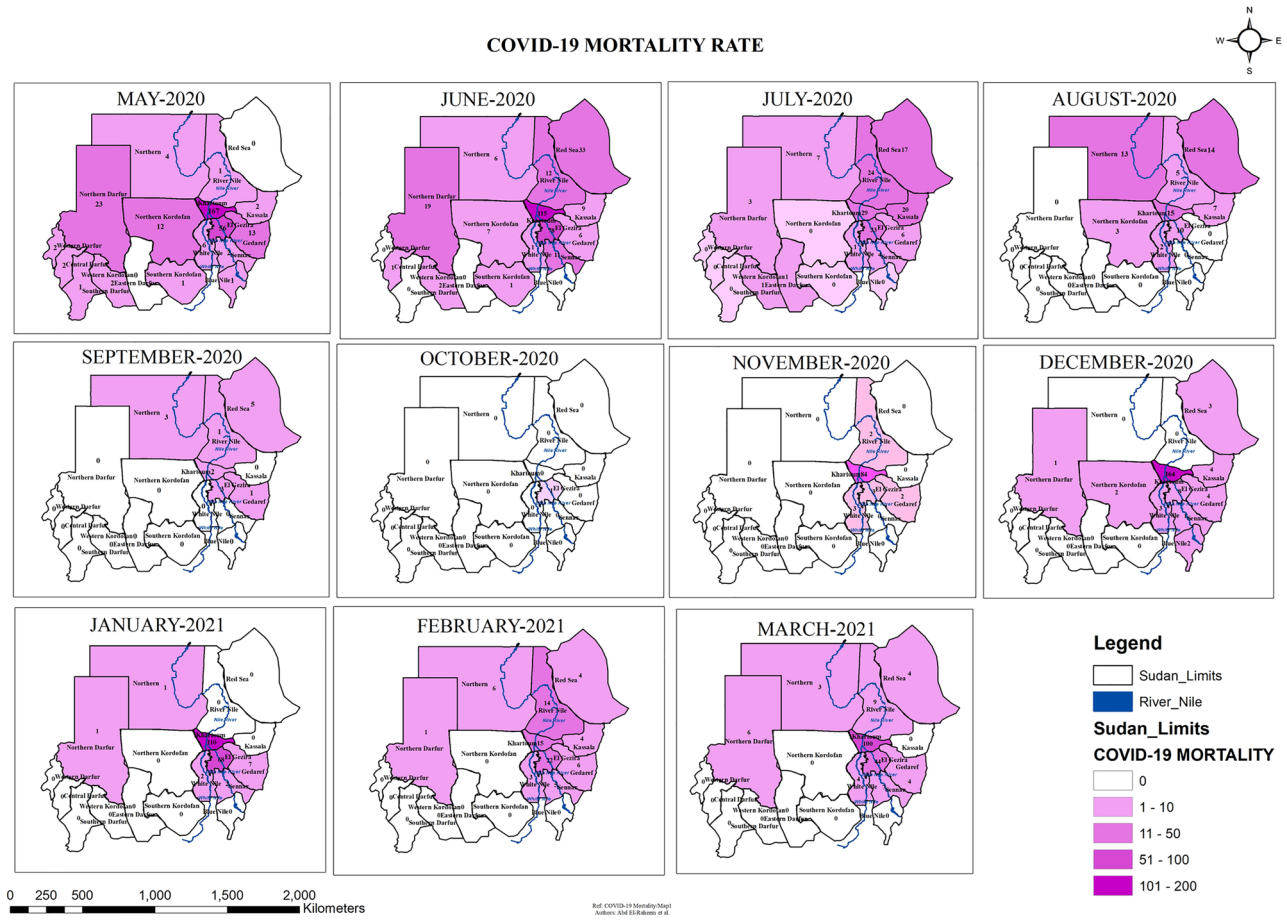
COVID-19 Mortality rate across the states of Sudan May 2020-March 2021 developed through ArcGIS 10.8 by Abd El-Raheem et al. (Shp. files of the base maps https://www.diva-gis.org/gdata).

Mortality rate across the different states was studied (Fig. 3). The monthly mortality cases of COVID-19 were presented spatially. Mortality rate had differed from one state to another and from one month to the other (Fig. 3). Interestingly, in the surge of May and June-2020, the mortalities were prevalent in almost all the states. In May-2020, Khartoum, El Gezira, Northern Darfur and Northern Kordofan had the highest mortality rates (167, 56, 23 and 12 dead cases/month respectively). In June-2020, mortality was the highest in Khartoum, El Gezira and Northern Darfur (115, 78 and 19 dead cases/month respectively). The mortality rate was zero in October-2020 in 17 states of Sudan consistent with the infection rate. Only one state (El Gezira) had reported mortalities during October-2020 (one dead case). During the surge of December-2020 and January-2021; mortalities were centered in the middle states (Khartoum and El Gezira). About 10 states had zero mortalities in the surge of December and January. In March-2021, mortality rates were rising again; especially in Khartoum State (100 dead cases/month). Figure 3 illustrates the details of the mortality trend in Sudan across the different months.

### Cumulative COVID-19 mortality across the States of Sudan

**Figure 4:**
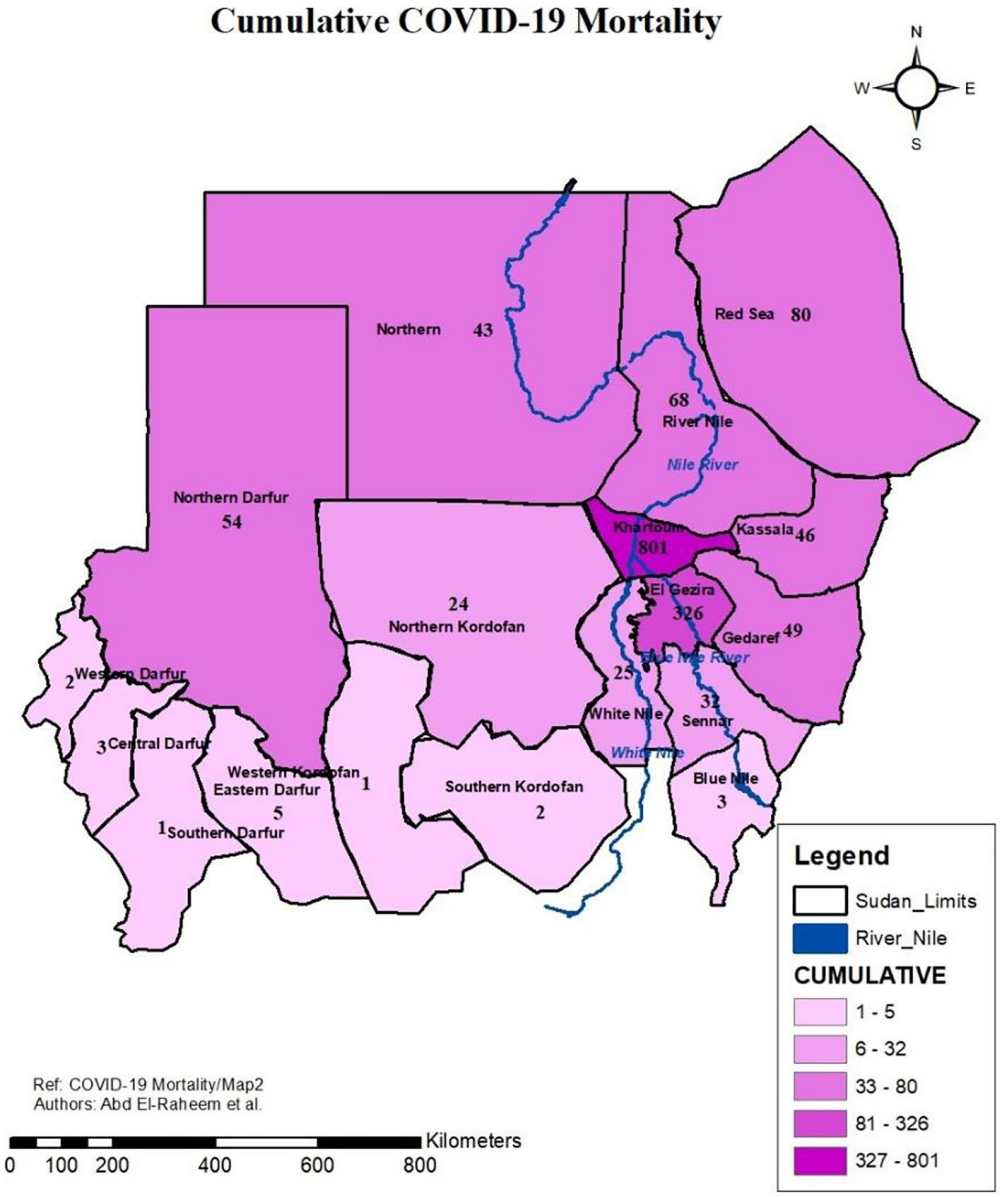
Cumulative COVID-19 Mortality across the Sudan States developed through ArcGIS 10.8 by Abd El-Raheem et al. (Shp. files of the base maps https://www.diva-gis.org/gdata).

Cumulative COVID-19 mortality map (Fig. 4) showed the overall mortality in Sudan at state level. The states with the highest cumulative mortality were Khartoum and El Gezira (801 and 326 dead cases respectively). While, the lowest mortalities were in Western Kordofan and Southern Darfur (1 dead case each). In general, the western and southern states of the country had the lowest mortality rates. While, the middle states (Khartoum and El Gezira) had the highest mortalities. Northern and eastern states had lower mortalities than the middle states, yet, higher than the western and southern states (Fig. 4).

### Prevalence of COVID-19 infection in Sudan

**Figure 5:**
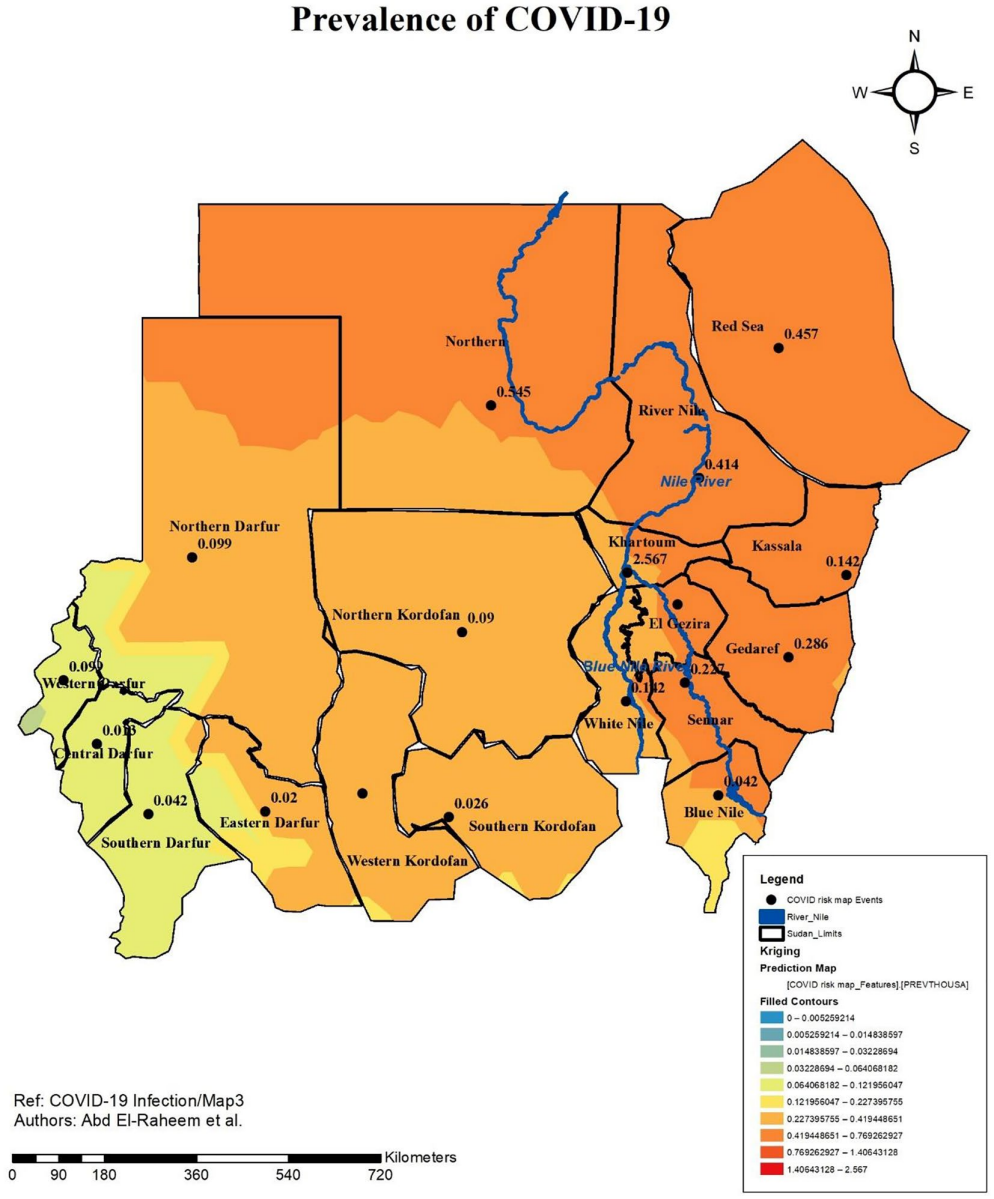
Risk Map of COVID-19 Infection in Sudan developed through ArcGIS 10.8 by Abd El-Raheem et al. (Shp. files of the base maps https://www.diva-gis.org/gdata).

Prevalence of COVID-19 infection in Sudan was obtained. The number of cases per thousand populations were calculated for each of the states of Sudan to develop the risk map (Fig. 5). Areas with the highest risk were the center and the east parts of the country. Khartoum State had the highest prevalence (2.6 cases/1000 populations). While, the areas with the lowest risk were the western parts of the country; with Western Darfur having the lowest risk of COVID-19 infection (prevalence = 0.09 cases/1000 populations). Figure 5 presented the risk map of COVID-19 in Sudan.

### Correlation between COVID-19 infection and mortality trends over time

**Figure 6:**
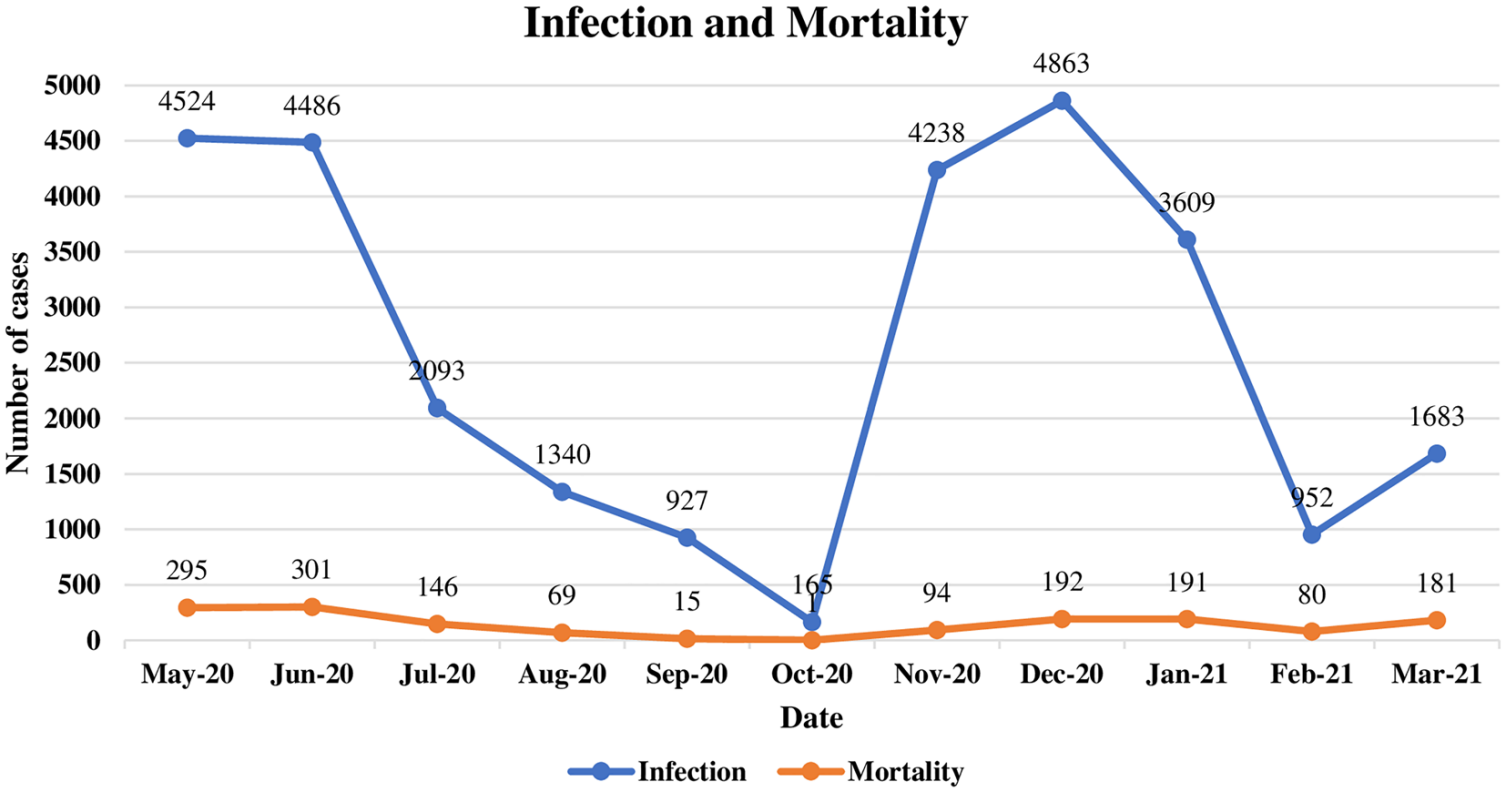
Correlation between COVID-19 infection and mortality over time (May, 2020–March, 2021).

Infection and mortality rates were studied together to find the correlation between these rates. Strong positive correlation between infection and mortality (Pearson’s correlation = 0.789, *p*-value = 0.004) was found in Fig. 6, the peaky nature of COVID-19 infection is clear; showing two peaks and the third peak was starting. Infection rate was high in May and June-2020, then started decreasing until October-2020 (lowest infection rate). During the peak of May 2020, mortality accounted for 6.5% of the cases (295/4524). After that, infection had started in increase in November, reaching a peak in December-2020.On the peak, COVID-19 mortality was 3.9% (192/4863). The December 2020 peak was then decreased in February-2021. In March, the infection rate had started to rise. Mortality trend was less peaky; mortality rates were much lower than the infection rates. Even though, increases in mortality rates were apparent in Fig. 6 consistent with the peaks of infection.

## Discussion

One of the chief qualities of epidemics is their spatial spread, “a characteristic which mainly depends on the epidemic mechanism, human mobility and control strategy”^20^. On global perspective, Africa generally was considered an “L-L” area, meaning that the areas with low number of deaths were close to other locations that had also a low number of deaths^21^. The overall burden of COVID-19 in the African continent is relatively low compared to Europe, Asia and the Americas. Even though, many African countries are poor and the early control measures; as isolation, quarantine and social distancing might not work as efficiently as in China^17^. The burden of COVID-19 disease in Africa was the greatest in Djibouti, Tunisia, Morocco and Algeria. This might be because of the testing capacities of these countries enabling them to conduct relatively more tests and detect more COVID-19 cases^17^. From the results of this study, it was apparent that there was a spatial relationship to the distribution of COVID-19 cases during the pandemic months in Sudan. That was the case in China; Xie et al. reported that the COVID-19 distribution of cases was changing through time but not fundamentally^22^. This meant that the pattern of infection in high prevalence areas and the low prevalence areas have not totally changed and only the number of cases differed through time. Some states of Sudan had experienced higher number of cases than others; specifically, Khartoum state and Gezira state. Additionally, it was also explicit that there have been temporal fluctuations to COVID-19 cases as variable numbers of cases have been documented through the months with a peaky nature. The peak infection rate (wave) of December 2020 presented in our study was the highest in Sudan comprising a monthly cumulative cases of 4863 cases. This was consistent with the reported rates in Africa^15^. Since the emergence of the pandemic, coronavirus transmission had been prevented and reduced by early detection. Regions with a fewer number of performed tests were unable to adequately to detect all the cases. Early detection of COVID-19 infection had been implemented in many countries in Africa such as Ivory Coast. In January 2020, the country started implementing checks for COVID-19 symptom at airports as well as, restriction of travels from China^18^. In Sudan, such procedures were initiated in March, 2020. When data reported by the ministry of health were studied, a major variation across the states was evident. The rational interpretation for the apparent variability in disease prevalence within different states in Sudan can be related to numerous factors. Social habits (greetings habits, ease to touch another person, kissing etc.) could play a part in augmenting the exposure of the population, the presence of “super-spreaders " or a slow execution of measures aimed to stop the spread of infectious disease such as social distancing and closure of public areas. Many studies have supported the link between environmental factors including air pollution, temperature, humidity, and corona virus disease susceptibility and severity^23–25^. Increased humidity was reported as a factor that had reduced the transmission rate of COVID-19^23^. Sudan is a tropical country, the climate humidity increases in the autumn season (between July and September), during this period, the COVID-19 infection rate was the lowest (Fig. 6). Reduced climate temperature in Sudan occur in the winter season between November and February, during this period, Sudan experienced a wave of COVID-19 pandemic with high infection rate. There was some evidence supporting that an association was found between high temperature and low severity of COVID-19. In Sudan, the situation was contradictory to that evidence, as the infection rate was the highest during May and June; the summer season. Similarly, to the outbreak patterns in Hong Kong, Australia, Malaysia and South Africa. High infection rates were reported in these countries despite the heat^16^. Not to forget that, the population density was one of the influencing factors for increased rate of COVID-19 infection^23^. In Sudan, as illustrated in Fig. 2 the highest cumulative infection was reported in Khartoum State which was the most populated state in Sudan. In general, the prevalence of COVID-19 in Sudan was relatively low in the southern and western states, while infection rate was relatively high in the middle, eastern and northern states. These spatial variations may need to be further studied to find out all the influencing factors. The mortality rates for patients with COVID-19 showed a significant variation across the different states of Sudan. COVID-19 prevalence and mortality rates were estimated to be higher in middle states of Sudan (e.g., Khartoum, El Gezira State) followed by the Northern states. These results could be attributed to the inequality and variations in health systems and medical equipment coverage, affordability, and accessibility to healthcare services. For example, some states struggle to perform adequate COVID-19 tests. Securing medication shortages is a public health issue, along with providing healthcare facilities to meet the needs of the community. Infected cases had to travel to urban areas to get good healthcare access^18^. This was the case in Sudan, almost all the states had very poor healthcare systems and many people had to urbanize to Khartoum state in order to get better healthcare. Prediction models were developed to predict urbanization based on socioeconomic factors and natural environmental factors^26^. Shortages in medication supply in Sudan might have affected the mortality rate in Sudan. These shortages have high negative impact especially during COVID-19 crisis^27^. In addition, the difference in epidemiological surveillance and detection capabilities can be related to significant variations in numbers among different states. As illustrated in Fig. 6, the mortality rate was positively correlated (*p* < 0.01) to the infection rate but not as high. In the wave of May 2020, COVID-19 mortality accounted for 6.5% of the cases in Sudan. However, in the peak of December 2020 this percentage had decreased (3.9%). However, in Sub-Saharan Africa the percentage of mortality was relatively lower; 2.4% deaths per total cases reported^28^. The healthcare system had a role in these reported mortality rates. The healthcare system in Sudan was not wide enough to combat the waves of the pandemic. A study was done in Khartoum, Sudan which defined the radius of the healthcare services of an isolation center and the number of cases that were covered^29^. Wider similar studies might provide a clearer picture about the healthcare coverage in Sudan. In areas where mortality rates are high in Sudan, there was a crucial, need to conduct more tests for identification of infected cases at early stages in order to enable authorities for a proper response in monitoring and controlling the transmission of the disease. As a way for humans to combat this infection, vaccination was highly encouraged^30,31^. Till May 2021, above 30 countries in Africa had less than 1% COVID-19 vaccination coverage^31^. The average vaccination coverage in the African continent was 2.5% only^31,32^. Studies similar to this type of spatial presentation could become a guide for vaccination strategies^21^. As hypothesized, the population behaviors such as mobilization served as a superior measure for justifying the spreading of COVID-19 over time and space^12^, our study was lacking this property of studying the mobility. This study was not without limitations; all the data were obtained from the records of the Sudan federal ministry of health which contained the number of cases tested in governmental laboratories only. All cases diagnosed in private laboratories were not included in the reports. This might lead to under-estimation of the true status.

## Conclusions

The western and southern states of the country had the lowest COVID-19 infection and mortality rates. While, the middle states (Khartoum and El Gezira) had the highest infection rates and mortalities. During the autumn season (July–September), the infection rate was decreased due to increased humidity. In Sudan, the peaky pattern of COVID-19 was prevalent in both the summer and winter seasons. These seasons witnessed increased rates of infection (waves). Therefore, researches are needed to meticulously study the climatologic impact on the rate of COVID-19 infection. Strong positive correlation between the rate of infection and mortality was found, this is explained by the presence of limited healthcare services that were not enough to keep a steady rate of mortality. Encouraging the expansion of the healthcare services is important in order to reduce the mortality rates during the waves of the pandemic (Supplementary Information S1).

## Supplementary Information

Supplementary Information.

## Data Availability

All data generated or analysed during this study are included in this published article [and its supplementary information files].

## Abbreviations

WHO: World Health Organization
COVID-19: Coronavirus disease 2019
GIS: Geographic information system
